# Collaboration in action: Protocol development across the MRC National Mouse Genetics Network

**DOI:** 10.1016/j.xpro.2026.104898

**Published:** 2026-10-15

**Authors:** Debora Bogani, Linus F. Reichenbach, Jillian L. Barlow, Karen Blyth

**Affiliations:** 1 Mary Lyon Centre at MRC Harwell, Didcot, OX11 0RD Oxfordshire, UK; 2 Cancer Research UK Scotland Institute, G61 1BD Glasgow, UK; 3 Centre for Blood Research, York Biomedical Research Institute, Department of Biology, University of York, Heslington, York YO10 5DD, UK; 4 NIHR, Leeds-York Biomedical Research Centre, Leeds, UK; 5 School of Cancer Sciences, University of Glasgow, G61 1QH Glasgow, UK

## Abstract

Collaboration and multidisciplinary approaches are key factors in the advancement of scientific research. This Backstory introduces the guiding philosophy and the collaborative nature of the MRC National Mouse Genetics Network (NMGN) and serves to set the foundation for the contents of this *STAR Protocols* focused collection. The collection brings together protocols and Primers that highlight methodological advances in mouse genetics, disease modelling, phenotyping, data integration, and related experimental approaches developed across the NMGN. Together, these contributions illustrate how distributed expertise, shared infrastructure, and coordinated protocol development can strengthen reproducibility, support responsible use of *in vivo* models and emerging alternatives, and accelerate translational biomedical research.

### A working collaborative model

This *STAR Protocols* collection, in collaboration with the MRC National Mouse Genetics Network (NMGN), reflects the breadth of expertise within the UK research community, highlighting current advances in preclinical modelling and associated methodologies. It brings together contributions from multiple disciplines and illustrates how experimental approaches can be developed in ways that support both innovation and application.

It also highlights how the ethos of the network and the principles of the journal align in promoting greater reproducibility and rigor through shared expertise and collaboration.

The NMGN is a UK Research and Innovation initiative comprising multiple disease-focused clusters pursuing distinctive but complementary challenge-led research. The clusters have a specific focus on aging, cancer, congenital anomalies, early life neurological disorders, mitochondrial diseases, microbiome, developing tools to study haematopoietic lineages, and *in vivo* protein modulation via degron tagging. Two associated clusters are also integrated within this framework and focus on genome-engineering horizon scanning and *in vivo* models of dementia and neurodegenerative diseases. The Mary Lyon Centre at MRC Harwell (MLC) acts as a central hub for the network, focused on generating and robustly phenotyping mouse strains, supporting pre-clinical studies, and delivering training. Across the network, expertise spans the development of genetic mouse models as well as translational and clinical research, creating clear pathways toward clinical application. Importantly, the clusters are not siloed, but rather interact to capitalize on opportunities for cross-disease insight, recognizing that shared mechanisms often underpin diverse conditions. The NMGN is also overseeing the development of a data platform designed for the collection, analysis, and release of network data and is ready to receive any relevant dataset from the preclinical and clinical research community. The entire structure is designed to maximize the impact of research outcomes across disciplines and disease areas.

The NMGN was established with a clear vision: to bring together diverse communities of researchers working at the interface of genetics, disease modeling, and translational biology and to provide a national framework for collaboration, resource sharing, and innovation. The distributed nature of the network is one of its core strengths. By bringing together *in vivo* biologists, clinicians, data scientists, geneticists, and technologists—and by embedding animal facilities and national infrastructure at its core—the network creates an environment in which new approaches can be developed, interrogated, and refined through collective expertise.

This collaborative model naturally supports the development and evolution of experimental methods. A new approach is not confined to a single research group; instead, it is shared across the network, where it can be adapted in different contexts. The use of home cage monitoring protocols developed at the MLC by multiple clusters for the identification and analysis of different lifecycle phases and phenotypes is one example of such a strategy. This process not only supports consistency in application but also encourages innovation, as methods are shaped by diverse perspectives and experimental needs. In this way, protocols emerge not as static outputs, but as evolving tools that reflect contributions from across the network. The establishment and maintenance of a new germ-free facility is a further example provided in this collection.^1^

The MLC plays an important role within this ecosystem. As a hub for *in vivo* and molecular workflows, the center is closely integrated with the activities of the clusters. Methods are both developed within and exchanged with the center, creating a two-way flow of knowledge and practice. This exchange provides an opportunity to align protocols with established operational standards, including those relating to animal welfare, colony management, and genetic integrity, while also enabling new approaches developed across the network to inform and enhance centralized practices. The center’s expertise and infrastructure therefore contribute to consistency and reliability while remaining part of a broader, distributed system of methodological development.

Importantly, this process is not only technical in nature but reflects a collaborative ethos that underpins the network. Researchers do not work in isolation but in partnership, with an understanding that progress is strengthened by multiple perspectives and disciplines. For *in vivo* specialists, this may involve testing new models or assays across different facilities (the network funded, among others, a project to assess the microbiome in mice across partner facilities^2^), ensuring that approaches are applicable in varied settings. For molecular and cell biologists, it may involve the joint development and optimization of genetic,^3^ imaging,^4^ or analytical techniques across laboratories.^5^^,^^6^ At each stage, methods benefit from shared input, transforming them from locally developed approaches into broadly applicable tools, also partially aiding the pursuit of higher-risk projects with wide application potential.^7^ This collective development is particularly valuable in addressing complex biological and clinical questions as exemplified in Watt et al.^9^ in this collection, describing mouse models of cancer, where integrating insights across systems and scales is essential to advancing understanding and supporting translation into therapeutic strategies.

The Primers and protocols gathered in this collection reflect the cross-disciplinary expertise that has coalesced the network and its collaborative approach. They illustrate not only state-of-the-art methodological development but also the value of working across institutional and disciplinary boundaries. For the wider scientific community, these protocols offer more than technical guidance—they represent approaches shaped by a shared commitment to using *in vivo* models and modern alternatives responsibly, to address complex questions relevant to the network’s disease areas and applicable to a much broader context.

This collection also highlights a broader principle. In an era where concerns around reproducibility and transparency in biomedical research are increasingly prominent, the NMGN demonstrates how a structured, collaborative model can respond to these challenges.^8^

This distributed approach is particularly important in the study of complex clinical problems, where biological systems are multifactorial and difficult to model in a single setting. Methods that have been developed and applied across multiple environments such as those optimized for breeding and phenotyping of axenic mice,^1^ or the standardized embryo dissection and phenotyping,^8^ are better positioned to capture this complexity, supporting more robust insights into disease mechanisms and increasing the likelihood that findings will inform advances in diagnosis and therapeutic development.^9^

Within this framework, established infrastructures such as the Mary Lyon Centre contribute to maintaining high standards through recognized practices and shared expertise, while operating alongside the contributions of network partners. Together, this combination of distributed innovation and coordinated support enables the development of approaches that are both flexible and reliable. Centralizing technologies and phenotyping platforms across disease areas creates opportunities to develop novel approaches and extend their application to new fields. A clear example is the application of well-established phenotyping tests to the study of aging—not a disease in itself, but a process widely recognized as driving the decline of multiple organ systems and their eventual progression into disease states.

For this *STAR Protocols* collection, we have collected protocols and Primers from authors across the network that can be read as more than isolated technical contributions. They are the outputs of a community working at scale, shaped by a shared commitment to methodological development and collaborative problem-solving, optimizing the best use of *in vivo* models in integrated platforms that include the latest developments in alternative methods in close alignment with the recent government white paper on replacing animals in science.^10^ They demonstrate how national infrastructure and distributed expertise can support new ways of developing, refining, and applying experimental approaches, particularly in the context of complex and interconnected disease landscapes.

In bringing this collection together, members of the NMGN provide not only practical tools for the scientific community but also an example of how collaboration and coordinated infrastructure can support the development of methods with relevance across multiple research areas and applications. The numerous contributions offered by NMGN’s partners to the recent Parliamentary Office of Science and Technology POSTnote on “technology alternatives to animals in life sciences research”^11^ as well as a recent editorial by the network’s Director Owen Sansom^8^ also highlight the strong engagement of this community with current national and international policy development in this space.

### Looking forward

As biomedical research continues to evolve, the NMGN is well positioned not only to respond to emerging challenges but also to contribute to shaping future approaches to experimental science. The strength of the network lies in its ability to integrate new technologies within a collaborative and well-connected framework, ensuring that innovation is supported by shared expertise and practical implementation.

A key area of development is the incorporation of rapidly advancing technologies, including single-cell and spatial omics, high-resolution and longitudinal imaging, and data-driven approaches such as artificial intelligence and machine learning. These technologies are expanding the scale and depth at which biological systems can be studied, while also creating new opportunities for integrating data across different levels of organization. Within the network, their adoption is accompanied by collaborative development and shared learning, enabling methods to be adapted and applied across different research contexts. The new network-commissioned and developed data platform will soon bring together information from all clusters and relevant external sources in one central location. Researchers and clinicians will be able to explore and analyze scientific data across their disease areas, making it easier to share findings and collaborate across disciplines. The goal is to unlock new insights and potential therapeutic approaches that might not be possible when working in silos. This will be a way to export the network’s model to the preclinical and clinical research ecosystem and provide a lasting legacy to the community and ultimately the patients.

At the same time, the network will continue to integrate with other national initiatives like the UK Human Functional Genomics Initiative and the Cancer Research UK Data Hub. New partnerships, capabilities, and areas of expertise will offer opportunities for collaboration and methodological exchange. The continued interaction between distributed research groups and shared infrastructures, including the Mary Lyon Centre, will support consistency while allowing flexibility in how approaches are implemented and developed locally. This balance will become increasingly important as experimental designs grow more complex and interdisciplinary.

Looking beyond the UK, the network also has the potential to inform wider approaches to collaborative research. The challenges associated with complexity, scale, and translation are shared internationally, and the model developed within the NMGN—combining distributed expertise with coordinated infrastructure—offers a framework that may be relevant in other contexts. In this sense, several of the *in vivo* protocols generated through the network represent not only practical resources but also examples of how collaborative method development can be organized and sustained.

More broadly, the collection reflects the network’s underlying approach: that high-quality science depends on methods that are clearly described, carefully developed, and adaptable across contexts. By capturing protocols shaped through collaboration, shared expertise, and iterative development, it contributes to ongoing efforts to strengthen experimental practice within the biomedical research community.

## Declaration of interests

The authors declare no competing interests.
